# 2-[^18^F]-FDG PET/CT Semiquantitative and Radiomics Predictive Parameters of Richter’s Transformation in CLL Patients

**DOI:** 10.3390/medicina60020203

**Published:** 2024-01-24

**Authors:** Domenico Albano, Anna Calabrò, Francesco Dondi, Francesco Bertagna

**Affiliations:** 1 Nuclear Medicine Unit, ASST Spedali Civili of Brescia, 25123 Brescia, Italy; anna_calabro@hotmail.it (A.C.); f.dondi@outlook.it (F.D.); francesco.bertagna@unibs.it (F.B.); 2 Radiological Sciences and Public Health Department, University of Brescia, 25123 Brescia, Italy

**Keywords:** 18F-FDG, CLL, PET/CT, Richter transformation, radiomics

## Abstract

*Background and Objectives*: Chronic lymphocytic leukemia (CLL) is the most common type of leukemia in developed countries, which can evolve into aggressive lymphoma variants, a process called Richter transformation (RT). The aim of this retrospective study was to analyze the role of 2-deoxy-2-[18F]fluoro-D-glucose positron emission tomography/computed tomography (2-[18F]-FDG PET/CT) and its semiquantitative and radiomics features in detecting RT and evaluate the impact on overall survival (OS). *Materials and Methods*: One hundred and thirty-seven patients with histologically proven CLL were retrospectively recruited. PET/CT images were qualitatively and semiquantitatively examined by estimating the main metabolic parameters (the maximum standardized uptake value body weight (SUVbw), lean body mass (SUVlbm), body surface area (SUVbsa), lesion-to-blood-pool SUV ratio (L-BP SUV R), lesion-to-liver SUV ratio (L-L SUV R), metabolic tumor volume (MTV), and total lesion glycolysis (TLG) and radiomics first- and second- order variables of the lesion with highest uptake. The role of these parameters in predicting RT and OS was analyzed. *Results*: One hundred and thirty (95%) PET/CT scans were positive, showing an increased tracer uptake at the site of disease, whereas the remaining 7 (5%) scans were negative. SUVbw, SUVlbm, SUVbsa, L-L SUV ratio, and L-BP SUV ratio were significantly higher in the RT group (*p* < 0.001 in all cases). Radiomics first- and second-order features were not significantly associated with RT. After a median follow-up of 44 months, 56 patients died; OS was significantly shorter in patients with RT than patients without RT (28 vs. 34 months; *p* = 0.002). Binet-stage, RT, and L-BP SUV R were shown to be independent prognostic features. *Conclusions*: Semiquantitative PET/CT parameters such as SUVbw, SUVlbm, SUVbsa, L-L SUV ratio and L-BP SUV ratio may be useful in discriminating patients with a high risk of developing RT, whereas Binet-stage, RT, and L-BP SUV R are also significant in predicting OS.

## 1. Introduction

Chronic lymphocytic leukemia (CLL) is one of the most frequent types of leukemia. It typically happens in elderly patients and has a highly variable clinical course characterized by the clonal proliferation and accumulation of mature CD5-positive B-cells within the blood and some organs, such as bone marrow, lymph nodes, and the spleen [1]. The primary leukemogenic event could involve multipotent and self-renewing hematopoietic stem cells [2]. For the diagnosis, it is fundamental to evaluate the blood counts, blood smears, and immunophenotyping of circulating B-lymphocytes, which helps recognize a clonal B-cell population carrying the CD5 antigen as well as typical B-cell markers [3]. Sometimes, this leukemia may evolve into more aggressive variants: the most common is diffuse large B-cell lymphoma, followed by Hodgkin lymphoma. This phenomenon is called Richter transformation (RT) and happens approximately in 2 to 10% of cases [4]. RT may happen also in other lymphoproliferative disease such as small lymphocytic lymphoma. Several genetic, clinical, and biologic features are associated with a higher risk of RT [4], but a shared consensus is still lacking. Some studies have demonstrated that the presence of B symptoms, lactate dehydrogenase (LDH) level, b2 macroglobulin the asymmetric rise of bulky lymph nodes, and some molecular features, such as TP53 interruption and immunoglobulin heavy chain variable somatic hypermutation, may predict RT [5].

An excisional lymph node biopsy is considered the gold standard for the detection of RT, but it is fundamental to recognize a priori the right lymph node to biopsy. In this setting, the research of alternative imaging technique with good accuracy in recognizing the aggressiveness and the risk of evolution of nodes has been highly considered. It is well known that 2-deoxy-2-[18F]fluoro-D-glucose positron emission tomography/computed tomography (2-[18F]-FDG PET/CT) is a non-invasive imaging that may help to recognize the optimal site for biopsy (usually the site with higher uptake) [6,7], but solid studies based on larger populations are scarce. Moreover, PET/CT may study the whole body with a unique scan helping detect any site of abnormal uptake. RT is a poor prognostic factor associated with a median survival of about 2–10 months [3,4]. The aim of this retrospective research was to study the role of 2-[18F]-FDG PET/CT and its semiquantitative and radiomics features in detecting RT and the possible impact on outcome in terms of overall survival (OS).

## 2. Materials and Methods

### 2.1. Population

Between January 2009 and June 2023, 137 consecutive patients with histologically proven CLL who underwent 2-[18F]-FDG PET/CT scans for suspicious RT were included. Suspicion of RT was done by hematologists considering physical examination and blood counts as first level, and radiological findings. Usually, patients with suspected RT had enlarged lymph nodes increasing in size and/or number at CT associated with clinical deterioration. Particularly, in 65 cases there was a dimensional numerical increase of lymph nodes; in 30 cases a worsening of anemia and/or thrombocytopenia and in 20 cases the appearance of symptoms and signs such as weight loss >10%, fevers for more than 15 days and a significant fatigue. In the remaining 22 patients, a combination of previous alterations was present.

Exclusion criteria were: (a) patients without a histological confirmation of CLL; (b) patients with less than 18 years at the time of PET/CT; (c) patients without at least 6 months of follow-up after PET/CT; (d) patients that did not perform biopsy after PET/CT.

In all patients, several epidemiologic parameters (age, gender), morphological features (size, B-symptoms, bulky disease), stage disease (according to Rai and Binet classification), clinical parameters (white blood cell count, hemoglobin level, platelet count, Ki-67 score, LDH and b2 microglobulin level), 2-[18F]-FDG PET/CT features, previous treatments, and follow-up data were recorded. LDH and b2 microglobulin levels were considered normal or not normal considering a specific cutoff of 245 U/L and 2.5 md/dL, respectively, as per reference range of our hospital.

In 60 cases, patients did not receive previous treatments, defined treatment-naive. In the other 77 patients: 15 were treated with rituximab-bendamustine; 23 with ibrutinib; 17 with FCR (fludarabine, cyclophosphamide, and rituximab) regimen; 10 with rituximab-bendamustine plus ibrutinib; 8 with FCR plus rituximab-bendamustine; 4 with FCR plus rituximab-bendamustine and ibrutinib.

### 2.2. 2-[18F]-FDG PET/CT Imaging and Interpretation

2-[18F]-FDG PET/CT scans were acquired on a Discovery ST or 690 PET/CT (GE, Milwaukee, WI, USA). Parameters for acquisition: 80 mA, 120 Kv for CT (no enhanced contrast); 2.5–3 min per bed-PET-step of 15 cm, 256 × 256 as matrix of reconstruction, 60 cm as the field of view. At least 6 h of fasting before radiotracer injection was suggested and the glucose level was verified before radiotracer injection and was lower than 150 mg/dL in all cases (average value 101 mg/dL). Then, 3.5 MBq/Kg of 2-[18F]-FDG was administered intravenously, and PET/CT studies were acquired after an uptake time of about 60 min. The acquisitions were extended from the skull base to the mid-thigh.

2-[18F]-FDG PET/CT scans were reviewed by two nuclear medicine physicians (DA, AC) both qualitatively and semi-quantitatively by measuring the following features of the lesion with highest uptake: maximum standardized uptake value corrected for body weight, corrected for lean body mass, and corrected for body surface area (SUVbw, SUVlbm, SUVbsa), lesion to liver SUVmax ratio (L-L SUV R), lesion to blood-pool SUVmax ratio (L-BP SUV R), metabolic tumor volume (MTV), and total lesion glycolysis (TLG). For the visual analysis, the reviewer knew patient anamnesis, and judged the scan positive in presence of any focal radiopharmaceutical uptake away from physiological distribution and. For semi-quantitative analysis, the reviewer created a region of interest (ROI) corresponding to the lesion with highest uptake and the different SUVmax values (SUVbw, SUVlbm, SUVbsa) were measured. For the SUV ratios, hepatic SUVmax was measured at the VIII hepatic segment using a round-shape ROI of 10 mm; instead, mediastinal blood-pool SUVmax was measured at the aortic arch with identical ROI and taking attention to avoid the vessel wall activity.

A specific software (Advantage Workstation 4.6, GE HealthCare, Haifa, Israel) was utilized for the extraction of metabolic volumes (MTV and TLG). We choose to apply an isocounter threshold method based on 41% from 2-[18F]-FDG PET images.

For the measurements of first- and second-order features, a SUV based automated contouring software LIFEx 6.30, (LIFEX, by the French Alternative Energies and Atomic Energy Commission (CEA), Gifsur-Yvette, Paris, France) were used.

In each patient, the lesion with the highest FDG uptake underwent a biopsy to confirm the RT or not. The mean interval between PET/CT and biopsy was 9 days (range: 2–20 days).

### 2.3. Statistical Analysis

All statistical analyses were carried out with the help of the software MedCalc version 19 (8400, Ostend, Belgium). The descriptive analysis of categorical and numeric features were performed, representing simple and relative frequencies, and mean, minimum, and maximum, respectively. Two-sample independent *t* test and the chi-square (χ^2^) test were performed to compare the main clinical, histopathologic, PET/CT variables, and radiomics first- and second-order features between CLL patients who developed RT and those who did not.

To find the best thresholds of variables to predict RT phenomenon, we used receiver operating characteristic (ROC) curve analyses (Table 1).

The survival curves (OS) were calculated using Kaplan–Meier method and a 2-tailed log rank test was used to compare the groups. To calculate the hazard ratio and its confidence interval, a Cox regression analysis was utilized. A *p*-value < 0.05 was considered as statistically significant. OS was measured from the time of 2-[18F]-FDG PET/CT scan until the time of death or up to the latest follow-up control.

## 3. Results

### 3.1. Patients Features

In the period included, 157 patients performed a 2-[18F]-FDG PET/CT scan with the indication of suspected RT. A total of 20 patients had negative PET/CT scans showing no site of increased radiotracer uptake, while the other 137 had positive scans. For our analyses, we focused upon patients with positive PET/CT. In the majority (*n* = 133, 97%) of cases, the site of the biopsy (corresponding to the site of highest uptake) was a nodal localization, while in the other 4 (3%) patients, the biopsies were executed to extra-nodal sites (maxillar sinus in 1 case, parotid gland in 1 case, stomach in 1 case, skin in 1 case). Among these 137 patients, there was a prevalence of male (*n* = 102, 74%). The median age was 62 years (range: 34–89 years). In many patients the disease stage was early, with no bulky disease, and no B symptoms were registered (Table 2).

Treatment protocol did not affect the FDG uptake of CLL; in the group that received chemoimmunotherapy, the mean SUVmax was 6 (range: 1.8–12), while in the group that received ibrutinib alone or as combination treatment, the mean SUVmax was 5.8 (1.5–11.6) (*p* = 0.456).

### 3.2. PET/CT Features

Considering PET/CT findings, all the scans had the presence of at least one site of increased 2-[18F]-FDG-uptake (higher than background) corresponding to nodal or extranodal disease. In 46 patients (34%), the subsequent biopsy confirmed the RT phenomenon, while the remaining 91 (66%) had a biopsy negative for RT.

In patients with RT, the final histological diagnosis showed DLBCL in 25 cases, HL in 12 cases, plasmablastic lymphoma in 7 cases, and mantle cell lymphoma in 2 patients. In patients without RT, the most common histological diagnosis was lymphocytic leukemia (*n* = 52), followed by necrosis (*n* = 5) and inflammation (*n* = 5).

In patients with positive 2-[18F]-FDG PET/CT, the mean SUVbw of the lesion that received biopsy was 6.3 (range: 1.2–30.1); the mean SUVlbm 5.7 (range: 0.9–27.81), the mean SUVbsa 1.7 (range: 0.4–9.39), the mean L-L SUV ratio 2.43 (range: 0.9–50), the mean L-BP SUV ratio 3.15 (0.9–55), the mean MTV 25,351 mm^3^ (range: 562–970,860), and the mean TLG 123,634 (range: 22–5,038,763).

Concerning the main epidemiological, clinical, and histological features, no significant differences were available between RT patients and not RT patients. Only the ki-67 scores were significantly higher in the RT group than the non-RT group (*p* = 0.003).

Among semiquantitative PET variables, all SUV values and SUV ratios were significantly higher in RT compared to non-RT patients, while for tumor burden features (MTV and TLG) no significant differences were present (Table 2).

Instead of concerning radiomics parameters, no parameter was significantly associated with the risk of RT.

### 3.3. Prognostic Role of PET/CT

After a median follow-up of 44 months (range: 2–140), death was registered in 56 patients (41%). The median OS was 31 months (range: 1–179 months) for the whole population; the median OS was 34 months (range: 1–159 months) in the non-RT group and 28 months (range: 1–179 months) in the RT group (*p* = 0.002). In univariate analysis, Binet stage, the RS phenomenon and L-BP SUV R were significantly correlated with OS (Table 3). Instead, all the other variables, including several metabolic PET/CT variables (SUVbw, SUVbsa, SUVlbm, L-L SUV R, MTV, and TLG), resulted in no association with OS, along with other clinical/histological parameters (gender, age, tumor size, presence of B symptoms, presence of bulky disease, LDH and b2 microglobulin levels, and Ki-67 score). Binet stage, RT and L-BP SUV R were confirmed to be independent prognostic factors also in multivariate analysis (*p* = 0.016, *p* = 0.025, and *p* = 0.028, respectively) (Table 3). In addition, radiomic features were not significantly associated with OS. Among the patients who died, 25 were treatment naive, while 31 received at least 1 therapy before PET (*p* NS).

## 4. Discussion

The risk of RT in CLL is usually between 2 and 10%, but it is directly associated with the clinical features of the patients. In our study we got a higher rate of transformation (34%). This is due to the fact that not all patients with CLL performed PET/CT, but only patients with high risk of RT. This risk is derived by hematologists according to blood samples, clinical condition, and radiological findings. For similar reasons, the survival rate of our population was low, also in patients without RT. Another potential explanation could be the fact that about 50% of patients were not treatment-naive but received previous treatments. The number of patients dead was higher in the not treatment-naive group, but this difference was not statistically significant.

Usually, CLL is a disease with low/faint FDG uptake, and the increase of uptake in selected localizations may be an indirect sign of aggressive evolution, typical of RT. This is due to the fact that the metabolic behavior at 2-[18F]-FDG PET/CT may be associated with the biological aggressiveness of CLL and the risk of RT in more aggressive variants [5].

In 2006, Bruzzi et al. [8] first investigated the potential impact of 2-[18F]-FDG PET/CT in the evaluation of RT in patients affected by CLL, recruiting 37 patients who underwent 57 2-[18F]-FDG PET/CT studies, and derived a cutoff of SUVmax of 5 as best to predict RT. Subsequent studies [9,10,11,12,13,14,15] confirmed the significant role of 2-[18F]-FDG PET/CT in this field, but they suggested different SUVmax thresholds (such as 5, 10, and 11). In our analysis, a SUVmax of 4.97 was calculated with the best compromise between specificity and sensitivity (65% and 70%, respectively; and AUC of 0.676). This finding is very close to previous studies [8,9,11,13]. Falchi et al. [11] analyzed the larger cohort of patients (*n* = 332) deriving the best threshold of SUVmax as 5. With this value, the negative predictive value was very high (92%) and the positive predictive value was very poor (51%). In our study, we derived a better compromise between sensitivity and specificity (Table 4).

One of the limitations of the literature published about this topic is the absence of metabolic parameters beyond SUVmax. SUV corrected for body weight is the most common utilized and famous parameter in the actual literature for assessing disease activity in different diseases, but it presents several limitations that reduce its application in the clinical practice [16,17]. In fact, its measurement is affected by patient features (weight, blood glucose level), technical parameters (acquisition and reconstruction protocols, scanner characteristics, risk of extravasation and/or residual activity in the syringe), and tumor size (risk of partial volume effect) [16]. For these reasons, other semiquantitative parameters were introduced and tested as alternative or complementary to SUVmax, like SUV corrected for body surface area and lean body mass, ratio between SUV of lesion and references organs (usually liver and/or mediastinal blood pool), and metabolic tumor burden variables (MTV, TLG).

However, for the detection of RT in CLL patients, only 1 paper [15] investigated all these parameters on 80 patients, demonstrating that all PET SUV-related parameters (SUVlbm, SUVbsa, L-L SUV ratio, L-BP SUV ratio) were significantly different between patients with and without RT (*p* < 0.001 in all cases). whereas MTV and TLG were not significantly associated with the risk of RT. Despite the fact that comparison with literature results may be affected by the heterogeneity of studies (such as the number of patients included), our findings were concordant with the fact that PET/CT SUV-related variables were useful in discriminating patients with a higher risk to evolve into RT. These results are strengthened by having been evaluated in a larger population (*n* = 137). Instead, metabolic tumor burden features (MTV and TLG) did not correlate with RT phenomenon. MTV and TLG are parameters that enable the simultaneous consideration of morphological and functional features of diseases expression, incorporating size and the metabolic activity at the same time [18]. Many articles showed a positive impact of TLG and MTV in the survival outcome of many oncological diseases, such as lymphoma [19,20]. However, for CLL patients, this evidence was not confirmed because for this kind of disease, the morphological features, such as size, are not crucial to predict RT. This was also confirmed by the non-association with tumor size and RT. This is a clear example of when metabolic findings are better than morphological findings.

First, in this study we evaluated the radiomic features in this field including the main first- and second-order variables. Radiomic features were promising variables introduced to limit the qualitative analysis and help the reader to have as much information as possible. It is a noninvasive way to derive quantitative parameters with different applications [21,22]. Despite many positive publications in several diseases, no previous studies on CLL patients exist; in our paper we demonstrated no significant association between first and second-order texture features with RT and prognosis. The explanation may be similar to MTV and TLG, due to the fact that radiomic features are also related to morphological features of disease (size, shape) and thus less related to functional findings. Besides, despite PET radiomics being a promising field, often the number of patients included in most papers is poor, and only few studies have performed in-depth validations. Therefore, the standardization seems to be fundamental for the future.

Instead, considering prognostic role of 2-[18F]-FDG PET/CT in CLL patients, we demonstrated that among clinical variables only Binet stage and RT were independently associated with OS, while among PET parameters only L-BP SUV ratio was significantly correlated with survival. Additionally, in this setting, MTV, TLG, and texture analysis features showed no prognostic impact. Pointoizeau et al. [23] found no prognostic role of PET parameters in a small cohort of patients (*n* = 28) studied at different centers. So far, our study was performed with a larger population, evaluating the prognostic role of PET/CT parameters in CLL.

The detection of RT is a prognostic factor well known in literature and also confirmed in our population [24,25,26]. The possible usefulness of 2-[18F]-FDG PET/CT in detecting RT may help better stratify the risk of transformation in CLL and enabling personalized follow-up and treatment. Our findings underline the point that 2-[18F]-FDG PET/CT may be useful in the clinical practice, providing fundamental functional information and indirectly expressing disease aggressiveness. Moreover, another advantage of PET/CT could be the guide in the choice of site of biopsy, which is crucial to recognize RT.

In the future, the development of new scanners and/or new radiotracers could be an advantage for the study of CLL patients. The increasing introduction into clinical practice of PET/CT scanners with silicon photomultiplier (SiPM) technology, new algorithms or large field of acquisition (“total body PET”) will likely lead to new advances in the field of functional imaging, but studies are desirable in this direction. Particularity, in CLL patients, no specific studies are currently available, but the reduction of time acquisition and the reduction of activity administered is without any doubts a benefit.

Regarding new radiophamaceuticals, only one article [27] studied the potential role of [68Ga]Ga-Pentixafor in CLL with promising results.

This article presents some limitations, such as the retrospective plan of the research and the limited sample number of the patients recruited, directly associated to the rarity of the condition. More works based on larger cohorts are mandatory to confirm or rebut our evidence.

## 5. Conclusions

In conclusion, with this study we have demonstrated that the semiquantitative PET/CT features (SUVbw, SUVlbm, SUVbsa, L-L SUV ratio, and L-BP SUV ratio) may be useful in discriminating patients with a high risk of developing RT, but not for MTV, TLG, and radiomic features. Moreover, Binet-stage, RT, and L-BP SUV R are significant in predicting OS.

## Figures and Tables

**Table 1:** Cutoff values of semiquantitative PET/CT parameters calculated with ROC curve analysis.

Parameter	Cutoff	AUC (95% CI)	*p*-Value	Sensitivity (95% CI)	Specificity (95% CI)
SUVbw	4.97	0.676 (0.591–0.753)	<0.001	70% (54–82)	65% (54–77)
SUVlbm	4.87	0.642 (0.556–0.722)	0.006	48% (33–63)	80% (71–88)
SUVbsa	1.41	0.641 (0.555–0.721)	0.006	56.5% (41–71)	72.5% (62–81)
L-L SUV R	1.9	0.722 (0.639–0.795)	<0.001	72% (56.5–84)	68% (57.5–77.5)
L-BP SUV R	3.5	0.679(0.594–0.756)	<0.001	50% (35–65)	82% (73–90)
MTV	2762	0.513 (0.426–0.599)	0.807	41% (27–57)	65% (54–75)
TLG	8638	0.560 (0.473–0.658)	0.241	72% (56.5–84)	42% (31.5–53)

AUC: area under curve; CI: confidence interval; SUV: standardized uptake value; bw: body weight; lbm: lean body mass; bsa: body surface area; L-L R: lesion to liver ratio; L-BP R: lesion to blood pool ratio; MTV: total metabolic tumor volume; TLG: total lesion glycolysis.

**Table 2:** Characteristics of the population.

	N (%)			
	All (*n* = 137)	CLL Not RT (*n* = 91)	RT (*n* = 46)	*p*-Value
Gender male:female	103:34	67:24	36:10	0.976
Age, median (range)	62	63	60	0.650
Treatment naive	60 (44%)	36 (40%)	24 (52%)	0.190
B symptoms	25 (18%)	15 (16%)	10 (22%)	0.250
Bulky disease	20 (15%)	12 (13%)	8 (17%)	0.665
Rai stage I:II:III:IV	37:64:21:15	25:40:14:12	12:24:7:3	0.360
Binet stage A:B:C	55:55:27	35:30:17	20:25:10	0.458
Mean tumor size hypemetabolic lesion mm	26.1	25.1	28.22	0.470
Increased LDH level	52 (38%)	33 (36%)	19 (41%)	0.575
Increased β2 microglobulin level	46 (34%)	28 (30%)	18 (39%)	0.180
Ki-67 score ≥ 30% (*n* = 75)	30 (22%)	14 (15%)	16 (35%)	0.003 *
Mean SUVbw	6.3	5.02	8.84	<0.001 *
Mean SUVlbm	5.7	4.1	7.05	<0.001 *
Mean SUVbsa	1.7	1.37	2.34	<0.001 *
Mean L-L SUV ratio	2.43	1.78	3.74	<0.001 *
Mean L-BP SUV ratio	3.15	2.41	4.64	<0.001 *
Mean MTV mm^3^	25,351	29,600	16,944	0.489
Mean TLG	123,634	126,110	118,736	0.934

CLL = Chronic lymphocytic leukemia; L-L SUV ratio = lesion-to-liver SUV ratio; L-BP SUV ratio = lesion-to-blood-pool SUV ratio; LDH = lactate dehydrogenase; MTV = metabolic tumor volume; RT = Richter transformation; SUV = standardized uptake value; SUVbsa = SUV body surface area; SUVbw = SUV body weight; SUVlbm = SUV lean body mass; TLG = total lesion glycolysis. * *p*-value significant.

**Table 3:** Univariate and multivariate analyses for the evaluation of OS.

	Univariate Analysis		Multivariate Analysis	
	*p*-Value	HR (95% CI)	*p*-Value	HR (95% CI)
OS				
Sex	0.456	0.545 (0.222–1.400)		
Age >65 y	0.700	0.798 (0.345–1.777)		
Stage disease	0.010 *	0.234 (0.014–0.300)	0.016 *	3.535 (1.271–9.826)
Treatment naive	0.250	0.530 (0.064–0.896)		
Tumor size	0.670	1.202 (0.246–3.654)		
β2 microglobulin increased	0.901	1.038 (0.450–2.254)		
LDH increased	0.333	1.245 (0.539–2.695)		
Bulky disease	0.256	1.149 (0.365–3.632)		
B symptoms	0.350	0.590 (0.054–0.846)		
Ki-67 score high	0.778	0.870 (0.312–2.391)		
Richter syndrome	0.019 *	0.540 (0.310–0.942)	0.025 *	0.600 (0.364–0.995)
SUVbw °	0.133	0.671 (0.399–1.145)		
SUVlbm °	0.086	0.615 (0.328–1.151)		
SUVbsa °	0.257	0.734 (0.419–1.286)		
L-L SUV R °	0.269 *	0.740 (0.439–1.274)		
L-BP SUV R °	0.008	0.491 (0.263–0.918)	0.028 *	0.368 (0.112–0.590)
MTV °	0.550	0.589 (0.230–1.601)		
TLG °	0.358	0.476 (0.122–1.305)		

OS: overall survival; HR: hazard ratio; CI: confidence interval; ° parameters divided using thresholds after ROC analysis; * *p*-value significant.

**Table 4:** Literature review about the role of 2-[18F]-FDG PET/CT in RT.

First Author	Year	Country	Design	N° Patients	RT (%)	SUVmax Threshold	Sensitivity (%)	Specificity (%)	PPV (%)	NPV (%)
Bruzzi JF [8]	2006	USA	R	37	27%	5	91%	80%	53%	97%
Conte MJ [13]	2014	USA	R	272	9%	5	nr	nr	nr	nr
Papajik T [12]	2014	Czech Republic	P	44	18%	nr	nr	nr	nr	nr
Falchi L [11]	2014	USA	R	332	29%	5	88%	47%	38%	92%
Mauro FR [9]	2015	Italy	R	90	19%	5	87%	71%	51%	94%
Michallet AS [10]	2016	France	P	240	10%	10	91%	95%	29%	99%
Mato AR [14]	2019	USA	R	57	14%	5	71%	4%	16%	33%
Albano D [15]	2020	Italy	R	80	22.5%	9	67%	90%	67%	90%
Present	2024	Italy	R	137	34%	4.97	70%	65%	62%	75%

N°: number; R retrospective; P prospective; PPV: positive predictive value; NPV: negative predictive value; nr not reported.

## Data Availability

Data available on request due to privacy/ethical restrictions.
